# *Sedum kamtschaticum* Exerts Hypnotic Effects via the Adenosine A_2A_ Receptor in Mice

**DOI:** 10.3390/nu16162611

**Published:** 2024-08-08

**Authors:** Yeon-Soo Kim, Bo Kyung Lee, Cha Soon Kim, Young-Seob Lee, Yoon Ji Lee, Kwan-Woo Kim, Dae Young Lee, Yi-Sook Jung

**Affiliations:** 1 Department of Pharmacy, Ajou University, Suwon 16499, Republic of Korea; dustn1596@ajou.ac.kr (Y.-S.K.); pfiffer@ajou.ac.kr (B.K.L.); 2 Research and Development Department, Genencell Co., Ltd., Yongin 16950, Republic of Korea; cskim@genencell.co.kr; 3 Development of Herbal Crop Research, Rural Development Administration (RDA), Eumseong 27709, Republic of Korea; youngseoblee@korea.kr (Y.-S.L.); yoong0625@korea.kr (Y.J.L.); swamp1@korea.kr (K.-W.K.); 4 BK21 FOUR KNU Creative BioResearch Group, School of Life Sciences, Kyungpook National University, Daegu 41566, Republic of Korea; dylee08@knu.ac.kr; 5 Research Institute of Pharmaceutical Sciences and Technology, Ajou University, Suwon 16499, Republic of Korea

**Keywords:** *Sedum kamtschaticum*, myricitrin, hypnotic effect, adenosine A_2A_ receptor, GABAergic neuron

## Abstract

Insomnia is a common sleep disorder with significant societal and economic impacts. Current pharmacotherapies for insomnia are often accompanied by side effects, necessitating the development of new therapeutic drugs. In this study, the hypnotic effects and mechanisms of *Sedum kamtschaticum* 30% ethanol extract (ESK) and one of its active compounds, myricitrin, were investigated using pentobarbital-induced sleep experiments, immunohistochemistry (IHC), receptor binding assays, and enzyme-linked immunosorbent assay (ELISA). The pentobarbital-induced sleep experiments revealed that ESK and myricitrin reduced sleep latency and prolonged total sleep time in a dose-dependent manner. Based on c-Fos immunostaining, ESK, and myricitrin enhanced the GABAergic neural activity in sleep-promoting ventrolateral preoptic nucleus (VLPO) GABAergic. By measuring the level of GABA released from VLPO GABAergic neurons, ESK and myricitrin were found to increase GABA release in the hypothalamus. These effects were significantly inhibited by SCH. Moreover, ESK exhibited a concentration-dependent binding affinity for the adenosine A_2A_ receptors (A_2A_R). In conclusion, ESK and myricitrin have hypnotic effects, and their underlying mechanisms may be related to the activation of A_2A_R.

## 1. Introduction

Sleep is a physiological process necessary for sustaining optimal brain function and overall health [1,2]. Sleep disorders, which arise owing to insufficient or excessive sleep and abnormal nocturnal movements, include insomnia, restless leg syndrome, and narcolepsy [3,4]. The most prevalent sleep disorder, insomnia, is defined by challenges in sleep onset or maintenance, along with recurrent awakenings coupled with the inability to return to sleep [1,4]. This condition is associated with cardiovascular diseases, diabetes, depression, and cognitive impairment [5,6,7,8,9]. Benzodiazepines are frequently used to treat insomnia; however, prolonged use is associated with notable adverse effects, such as dependence, daytime sedation, lethargy, fatigue, heightened fall risk, and cognitive dysfunction [10,11]. Consequently, novel therapeutic targets and drugs for insomnia must be urgently explored.

Sleep and wakefulness are governed by the neurotransmitters and neuromodulators released in different brain areas [12]. Acetylcholine from the basal forebrain (BF), orexin from the lateral hypothalamus (LH), histamine from the tuberomammillary nucleus, and serotonin from the dorsal raphe nucleus promote wakefulness. Conversely, sleep is promoted by GABA from the ventrolateral preoptic nucleus (VLPO), melatonin from the pineal gland, and adenosine from the BF [13,14]. Sleep can be induced via the activation of sleep-promoting neurons or inhibition of wake-promoting neurons [15]. Adenosine, a key neurotransmitter, is crucial for the regulation of the sleep-wake cycle by binding to its receptors, particularly the adenosine A_1_ receptor (A_1_R) and adenosine A_2A_ receptor (A_2A_R), which are implicated in sleep modulation [16]. However, current sleep medications do not target A_2A_R.

Recently, medicinal plants have garnered growing interest owing to their affordability and lower incidence of side effects relative to conventional medications [17,18]. Several dietary and plant supplements, such as ashwagandha (*Withania somnifera*) and rice bran alcohol extract, are already used in clinical settings [19,20]. *Sedum kamtschaticum*, a perennial plant native to Korea, China, and Japan, has been found to possess beneficial properties, including promoting blood circulation and exhibiting anti-inflammatory and antioxidant effects [21,22,23]. This plant also has anxiolytic and cognitive enhancement properties; however, its hypnotic effects have not been reported [22,23,24]. In the present study, pentobarbital-induced sleep behavior experiments were performed to determine whether the ethanol extracts of *Sedum kamtschaticum* (ESK) and its active compounds, desmanthin, myricitrin, and quercitrin (https://scienceon.kisti.re.kr/srch/selectPORSrchReport.do?cn=TRKO202300028127&dbt=TRKO, accessed on 20 June 2024) exhibit hypnotic effects. In addition, the mechanism underlying this hypnotic effect was explored.

## 2. Materials and Methods

### 2.1. Materials

Diazepam, which was used as the positive control, was purchased from Hanlim Pharmaceuticals Co., Ltd. (Seoul, Republic of Korea). Pentobarbital was purchased from Myungjin Pharmaceuticals Co., Ltd. (Seoul, Republic of Korea). Myricitrin and Luzindole (LUZ) were obtained from Tokyo Chemical Industry Co., Ltd. (Tokyo, Japan). ESK was provided by the Rural Development Administration (Jeonju, Republic of Korea). 8-cyclopentyl-1,3-dipropylxanthine (DPCPX), Flumazenil (FLU), 2-Pyridineethanamine dihydrochloride (PEA), TCB-2, and 2-(Furan-2-yl)-7-phenethyl-7H-pyrazolo[4,3-e] [1,2,4]triazolo[1,5-c]pyrimidin-5-amine (SCH58261) were purchased from Tocris Biosciences (Avonmouth, UK). Mouse anti-GAD67 antibody was purchased from Millipore (Burlington, MA, USA). Rabbit anti-c-Fos antibody was purchased from Cell Signaling Technology (Danvers, MA, USA). Quercitrin was purchased from Sigma-Aldrich (Seoul, Republic of Korea). Desmanthin was purchased from ChemFace (Wuhan, China).

### 2.2. Sedum kamtschaticum Extract Preparation

*Sedum kamtschaticum* was harvested from Eumseong, Chungbuk Province, Republic of Korea. A voucher specimen (voucher no. MPS002559) was deposited in the herbarium of the Department of Herbal Crop Research, National Institute of Horticultural and Herbal Science, Rural Development Administration, Eumseong, Republic of Korea. The dried sample was extracted via reflux extraction with fermented ethanol and water at 70 °C for 5 h and concentrated using a vacuum evaporator (Eyela, Tokyo, Japan). The product was dried in a spray dryer to produce ESK, which was used for the in vivo studies.

### 2.3. Animals

All animal experiments were conducted according to the guidelines of the Institutional Animal Care and Use Committee (IACUC) of Ajou University (approval number 2023-0006), and all animal handling and care procedures adhered to the Animal Care and Use Guidelines published by Ajou University. Animals were obtained from Orient Bio, Inc., (Seongnam, Republic of Korea). A total of about 700 eight-week-old male ICR mice were used for the pentobarbital-induced sleep experiment. For the immunohistochemistry (IHC) and enzyme-linked immunosorbent assay (ELISA) experiments, about 30 eight-week-old male C57BL/6 mice were used. Experimental animals were housed in cages, with 5 per cage, under a 12 h light cycle (8:00–20:00), with constant temperature (23 ± 1 °C) and humidity (60 ± 10%). Water and food were provided ad libitum.

### 2.4. Pentobarbital-Induced Sleep Experiments

A pentobarbital-induced sleep test was conducted to evaluate the sleep efficacy of ESK, desmanthin, myricitrin, and quercitrin. Pentobarbital, ESK, myricitrin, and quercitrin were dissolved in saline, whereas the agonists, antagonists of various receptors, and desmanthin were dissolved in 1% DMSO. Saline solution or 1% DMSO was administered to the control and vehicle groups, respectively. The control, vehicle, diazepam, and samples were orally administered 30 min before pentobarbital administration, while the agonists and antagonists of various receptors were orally or intraperitoneally administered 45 min before pentobarbital administration. Mice were housed in individual cages for testing. Sleep latency was measured as the time to the loss of the righting reflex, whereas total sleep time was defined as the time to restoration of the righting reflex.

### 2.5. Immunohistochemistry (IHC)

We performed IHC experiments to assess c-Fos immunoexpression in the VLPO after the administration of ESK and myricitrin. For this experiment, we primarily used antibodies that have already been validated in many studies through positive control and pre-adsorption tests [25,26,27,28]. Additionally, we performed a negative control test by omitting the primary antibody while retaining the secondary antibody. Through the results exhibiting no apparent fluorescent or DAB staining in these negative control sections, we confirmed the specificity of the primary antibody. After the treatment with SCH, ESK, myricitrin, and saline were administered at a 15 min interval. At 1 h after administration, mice were anesthetized with pentobarbital sodium (50 mg/kg, i.p.). The diaphragm of mice was then incised, and mice were perfused transcardially with saline. The brains were then extracted and preserved in 4% paraformaldehyde (PFA) at 4 °C for 24 h. After fixation, the brains were immersed in 30% sucrose solution for 48 h, embedded in optimal cutting temperature (OCT) compound, encased in OHP film, and stored at −80 °C for 24 h. Coronal sections of the frozen brains were sliced at 20 μm thickness using a Leica SM2400 microtome (Leica Microsystem Inc., Durham, IL, USA) and preserved at −20 °C until further processing.

For antigen retrieval, the brain sections were incubated at ambient temperature (25 °C) for 30 min and then immersed in 10 mM sodium citrate buffer. Following PBS washes, the sections were treated with 3% H_2_O_2_ for 5 min to inhibit endogenous activity. The sections were then blocked with 5% BSA in PBS containing 0.1% Triton X-100 for 1 h at 25 °C and with an anti-c-Fos primary antibody (1:500) (Cell Signaling Technology, #2250, Danvers, MA, USA) in blocking solution for 48 h. After washing with PBS, the sections were incubated with a biotinylated anti-rabbit secondary antibody (1:500) (Vector Laboratories Inc., BA-1000, Newark, CA, USA) in PBS for 1 h. Following more PBS washes, the sections were stained using the VECTASTAIN^®^ avidin–biotin complex (ABC) kit (Vector Laboratories, Burlingame, CA, USA) for 30 min, washed, and developed using the Dako Liquid 3,3′-diaminobenzidine (DAB) + Substrate Chromogen System kit (Agilent, Carpinteria, CA, USA) for 5 min. Brown c-Fos-positive neurons were examined under a light microscope.

After antigen retrieval, brain sections were blocked with 5% BSA in PBS containing 0.1% Triton X-100 for 1 h at ambient temperature (25 °C). The sections were incubated with anti-c-Fos rabbit antibody (1:400) (Cell Signaling Technology, #2250, Danvers, MA, USA) and anti-GAD67 mouse antibody (1:500) (Millipore, MAB5406, Burlington, MA, USA) at 4 °C for 48 h, washed with PBS, and incubated with goat anti-rabbit Alexa Fluor 568 (1:500) (Invitrogen Corporation, A11011, Carlsbad, CA, USA) and goat anti-mouse Alexa Fluor 488 (1:500) (Invitrogen Corporation, A11029, Carlsbad, CA, USA) for 1 h at ambient temperature (25 °C). Finally, the sections were stained with 4′,6-diamidino-2-phenylindole (DAPI; Molecular Probes, Eugene, OR, USA), washed with PBS, and examined using a confocal microscope.

### 2.6. Radioligand Receptor Binding Assay

The radioligand receptor binding assay of ESK to A_2A_R was performed using A_2A_R-overexpressing HEK-293 cells by Eurofins Pharmacology Services (St. Charles, MO, USA), as previously described. The following concentrations of ESK were tested: 0.1, 0.3, and 0.9 mg/mL. Inhibition or stimulation greater than 50% was deemed to indicate a substantial effect, whereas suppression or stimulation between 25 and 50% indicated a mild-to-moderate effect. Less than 25% suppression or stimulation was not considered significant.

### 2.7. ELISA Analysis

SCH 58261 was administered 15 min before ESK and myricitrin. One hour after all treatments, mice were anesthetized using sodium pentobarbital and transcardially perfused with saline. The brains of mice were subsequently extracted, and the hypothalamus was separated from the brain tissue and homogenized in PBS. The GABA levels in the tissue supernatants were determined using ELISA, according to the kit instructions.

### 2.8. Statistical Analysis

Experimental data are expressed as Mean ± SEM. Statistical analyses were performed using GraphPad Prism 8.0.2 software. To confirm that the data met the conditions for normal distribution, the Shapiro–Wilk test and Kolmogorov–Smirnov test were used. For data that did not meet the normality assumption, the non-parametric Kruskal–Wallis test was performed, followed by a one-way analysis of variance (ANOVA) using Tukey’s post hoc test. A *p*-value of less than 0.05 was considered to indicate statistical significance.

## 3. Results

### 3.1. ESK Exerts Hypnotic Effects in a Pentobarbital-Induced Sleep Model

**Figure 1:**
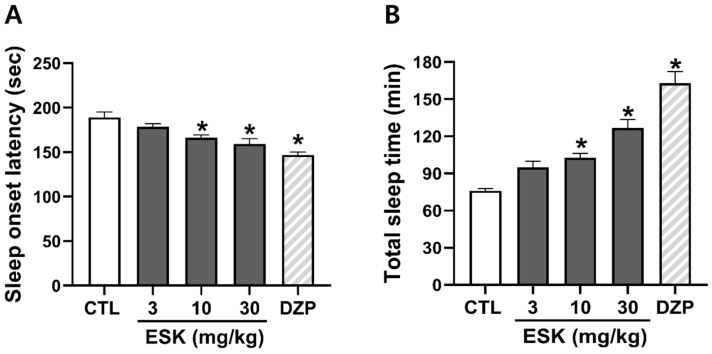
Hypnotic effect of ESK in a pentobarbital-induced sleep model. (**A**) Sleep onset latency and (**B**) total sleep time. ESK (3, 10, 30 mg/kg, p.o.) and diazepam (1 mg/kg, p.o.) were administered 30 min before pentobarbital (45 mg/kg, i.p.) administration. Data are presented as Mean ± SEM (*n* ≥ 5). * *p* < 0.05 vs. CTL. CTL, control; DZP, diazepam.

We explored the hypnotic effects of ESK using a pentobarbital-induced sleep behavior experiment. ESK (3, 10, 30 mg/kg) was found to decrease sleep onset latency (178.6 ± 3.4, 166.2 ± 3.2, 159.2 ± 6.0 s, respectively) and increase total sleep time (94.9 ± 5.0, 102.7 ± 3.7, 126.9 ± 6.7 min, respectively) in a concentration-dependent manner (Figure 1). DZP (1 mg/kg), a GABA_A_R-BDZ receptor agonist used as a positive control, markedly decreased sleep onset latency and increased total sleep time. In all subsequent experiments, an ESK concentration of 30 mg/kg was used, as this concentration had a similar effect to diazepam. Overall, these results suggest that ESK exerts hypnotic effects.

### 3.2. Hypnotic Effect of ESK Is Related to A_2A_R

**Figure 2:**
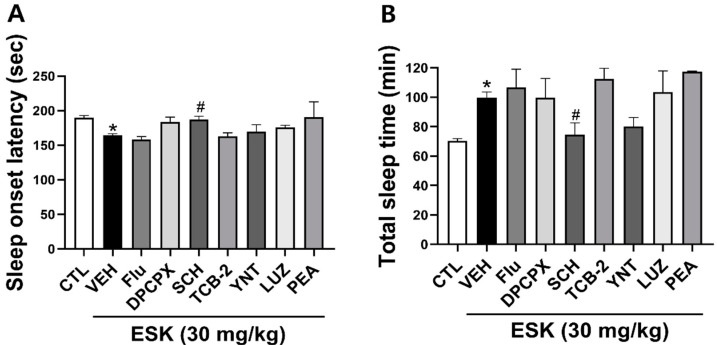
Effects of different agonists and antagonists on the hypnotic effect of ESK. (**A**) Sleep onset latency and (**B**) total sleep time. ESK (30 mg/kg, p.o.) was administered 30 min before pentobarbital (45 mg/kg, i.p.), while flumazenil (5 mg/kg, p.o.), DPCPX (5 mg/kg, p.o.), SCH (5 mg/kg, p.o.), luzindole (30 mg/kg, i.p.), PEA (150 mg/kg, i.p.), TCB-2 (10 mg/kg, i.p.), and YNT-185 (40 mg/kg, i.p.) was administered 45 min before pentobarbital. Data are presented as Mean ± SEM (*n* ≥ 4). * *p* < 0.05 vs. CTL; # *p* < 0.05 vs. VEH (ESK). CTL, control; DZP, diazepam; Flu, flumazenil; LUZ, luzindole.

To explore the mechanisms underlying the hypnotic effect of ESK, we used several drugs, including flumazenil (a GABA_A_ receptor antagonist), DPCPX (an adenosine A_1_ receptor antagonist), SCH (an adenosine A_2A_ receptor antagonist), luzindole (melatonin receptor_1_ antagonist), PEA (histamine H_1_ agonist), TCB-2 (a 5-HT_2A_ receptor agonist), and YNT-185 (Orexin_2_ receptor agonist). Among the many agonists and antagonists, only SCH significantly reversed the hypnotic effects of ESK. These findings suggest that the hypnotic effects of ESK involve A_2A_R (Figure 2).

### 3.3. ESK Influences Sleep-Wake Regulatory Regions in Mouse Brain

**Figure 3:**
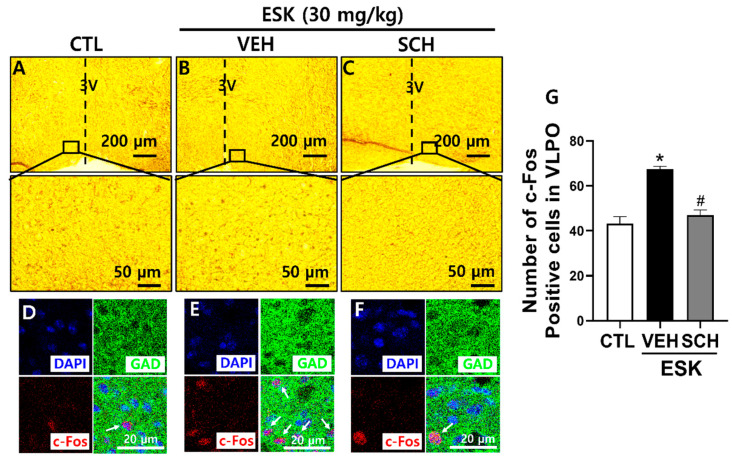
Effect of ESK on neuronal activity in VLPO. ESK (30 mg/kg, p.o.) was administered 1 h before brain extraction. SCH (5 mg/kg, p.o.) was administered 15 min before ESK administration. (**A**–**C**) Low-power and high-power microscopy images of VLPO. (**D**–**F**) Immunofluorescence images showing GAD67 (GAD, green), c-Fos (red), and DAPI (blue). White arrows indicate c-Fos positive cells. (**G**) The number of c-Fos positive cells. Data are presented as Mean ± SEM (*n* ≥ 3). * *p* < 0.05 vs. CTL; # *p* < 0.05 vs. VEH (ESK). CTL, control.

To explore whether the hypnotic effect of ESK is linked to sleep-regulating neurons in the mouse brain, IHC was performed. We examined the number of c-Fos neurons, an indicator of neural activity in the VLPO, which is a region that promotes sleep. Compared with the control, ESK increased neural activity in sleep-promoting VLPO GABAergic neurons. However, SCH reversed these effects (Figure 3). These findings suggest that ESK promotes sleep by activating VLPO GABAergic neurons via A_2A_R.

### 3.4. ESK Has Binding Affinity for A_2A_R

**Figure 4:**
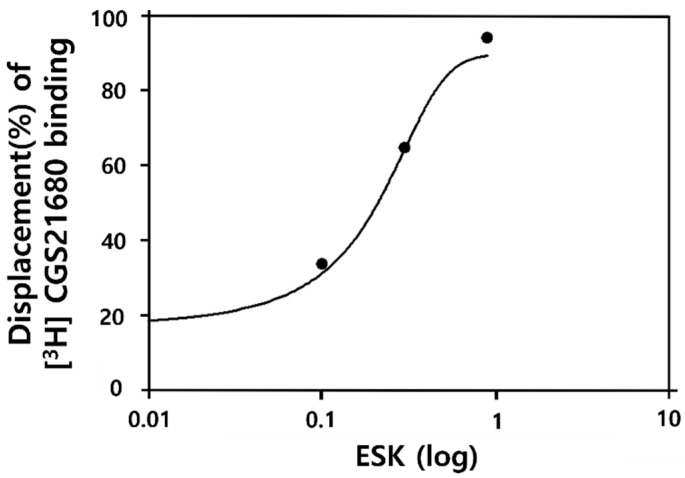
Binding affinity of ESK for A_2A_R. E ESK (0.1, 0.3, 0.9 mg/mL) was used for treatment, and binding was analyzed in A_2A_R overexpressing HEK293 cells. The coefficients of variation for the binding affinity of ESK at 0.1, 0.3, and 1 mg/kg were 16%, 5%, and 1%, respectively. Data are presented as mean ± SEM (*n* = 2).

To elucidate the hypnotic mechanism of ESK, we investigated its binding affinity to A_2A_R. ESK (0.1, 0.3, and 0.9 mg/mL) exhibited binding affinity for A_2A_R in a concentration-dependent manner (Figure 4): 0.1 mg/mL, 33.6%; 0.3 mg/mL, 64.7%; and 0.9 mg/mL, 94.0%. The IC_50_ was 0.21 mg/mL. These findings indicate that the hypnotic mechanism of ESK may involve A_2A_R.

### 3.5. ESK Compound Exerts Hypnotic Effect in a Pentobarbital-Induced Sleep Model

**Figure 5:**
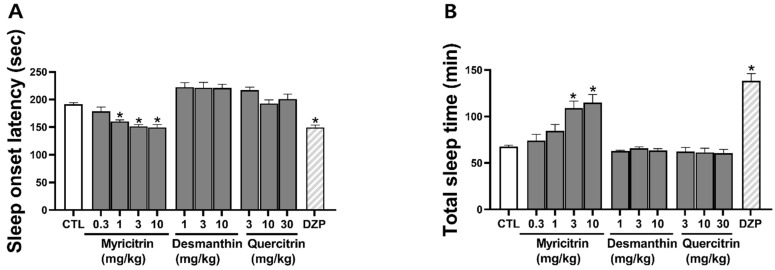
Effects of ESK compound in a pentobarbital-induced sleep model. (**A**) Sleep onset latency and (**B**) total sleep time. Myricitrin (0.3, 1, 3, 10 mg/kg, p.o.), desmanthin (1, 3, 10 mg/kg, p.o.), quercitrin (3, 10, 30 mg/kg, p.o.), and diazepam (DZP; 1 mg/kg, p.o.) were administered 30 min before pentobarbital (45 mg/kg, i.p.). Data are presented as Mean ± SEM (*n* ≥ 5). * *p* < 0.05 vs. CTL. CTL, control; DZP, diazepam.

To evaluate the hypnotic effects of the three ESK compounds, we used a pentobarbital-induced sleep model. Among the active compounds in ESK, only myricitrin exhibited a hypnotic effect; thus, desmanthin (1, 3, and 10 mg/kg) and quercitrin (3, 10, and 30 mg/kg) were not found to induce a hypnotic effect. Myricitrin (0.3, 1, 3, 10 mg/kg) was found to decrease sleep onset latency (178.8 ± 7.6, 160.3 ± 3.0, 151.6 ± 3.3, 149.8 ± 5.0 s, respectively) and increase total sleep time (67.4 ± 1.7, 74.2 ± 6.7, 84.4 ± 7.1, 109.1 ± 7.7 min, respectively) in a concentration-dependent manner (Figure 5). DZP (1 mg/kg), a GABA_A_R-BDZ receptor agonist used as a positive control, significantly decreased sleep onset latency and increased total sleep duration. In all subsequent experiments, a myricitrin concentration of 10 mg/kg was used, as this concentration had a similar effect to diazepam. These findings indicate that myricitrin may be responsible for the hypnotic effects of ESK.

### 3.6. Hypnotic Effect of Myricitrin Is Related to A_2A_R

**Figure 6:**
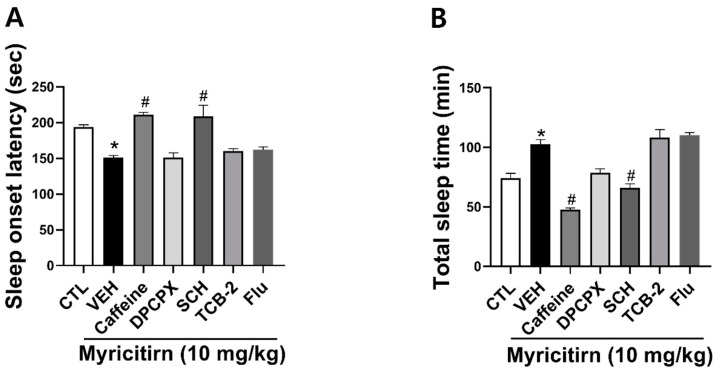
Effects of different antagonists and agonists on the hypnotic effect of myricitrin. (**A**) Sleep onset latency and (**B**) total sleep time. Myricitrin (10 mg/kg, p.o.) was administered 30 min before pentobarbital (45 mg/kg, i.p.), while DPCPX (5 mg/kg, p.o.), SCH (5 mg/kg, p.o.), TCB-2 (10 mg/kg, i.p.), Caffeine (10 mg/kg, i.p.), and Flumazenil (5 mg/kg, p.o.) were administered 45 min before pentobarbital. Data are presented as Mean ± SEM (*n* ≥ 4). * *p* < 0.05 vs. CTL; # *p* < 0.05 vs. VEH (Myricitrin). CTL, control; Flu, flumazenil.

To explore the mechanisms underlying the hypnotic effect of myricitrin, we used several compounds, including DPCPX (an adenosine A_1_ receptor antagonist), SCH (an adenosine A_2A_ receptor antagonist), TCB-2 (a 5-HT_2A_ receptor agonist), caffeine (an adenosine A_2A_ receptor antagonist), and flumazenil (a GABA_A_ receptor antagonist). Among the many agonists and antagonists, only SCH significantly reversed the hypnotic effects of myricitrin. These findings suggest that the hypnotic effects of myricitrin involve A_2A_R (Figure 6).

### 3.7. Myricitrin Influences Sleep-Wake Regulatory Regions in Mouse Brain

**Figure 7:**
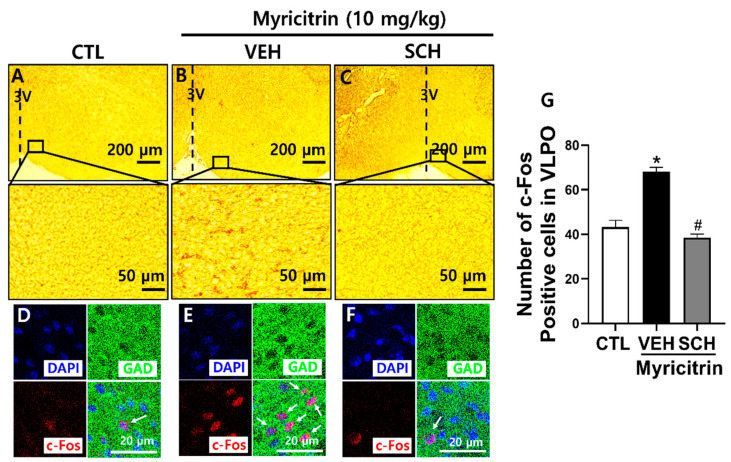
Effect of myricitrin on neuronal activity in VLPO. Myricitrin (10 mg/kg, p.o.) was administered 1 h before brain extraction. SCH (5 mg/kg, p.o.) was administered 15 min before myricitrin. (**A**–**C**) Low-power and high-power microscopy images of VLPO. (**D**–**F**) Immunofluorescence images showing GAD67 (GAD, green), c-Fos (red), and DAPI (blue). White arrows indicate c-Fos positive cells. (**G**) Number of c-Fos positive cells. Data are presented as Mean ± SEM (*n* = 5). * *p* < 0.05 vs. CTL; # *p* < 0.05 vs. VEH (Myricitrin). CTL, control.

To determine whether the hypnotic effect of myricitrin is linked to sleep-regulating neurons in mouse brains, we performed IHC. The number of c-Fos neurons, which is an indicator of neural activity, was examined in the VLPO, a sleep-promoting area. Myricitrin increased neural activity in the VLPO GABAergic neurons, thereby promoting sleep. However, SCH reversed this increase in neural activity (Figure 7). These findings indicate that myricitrin facilitates sleep by activating VLPO GABAergic neurons via A_2A_R.

### 3.8. ESK and Myricitrin Increase GABA Release

**Figure 8:**
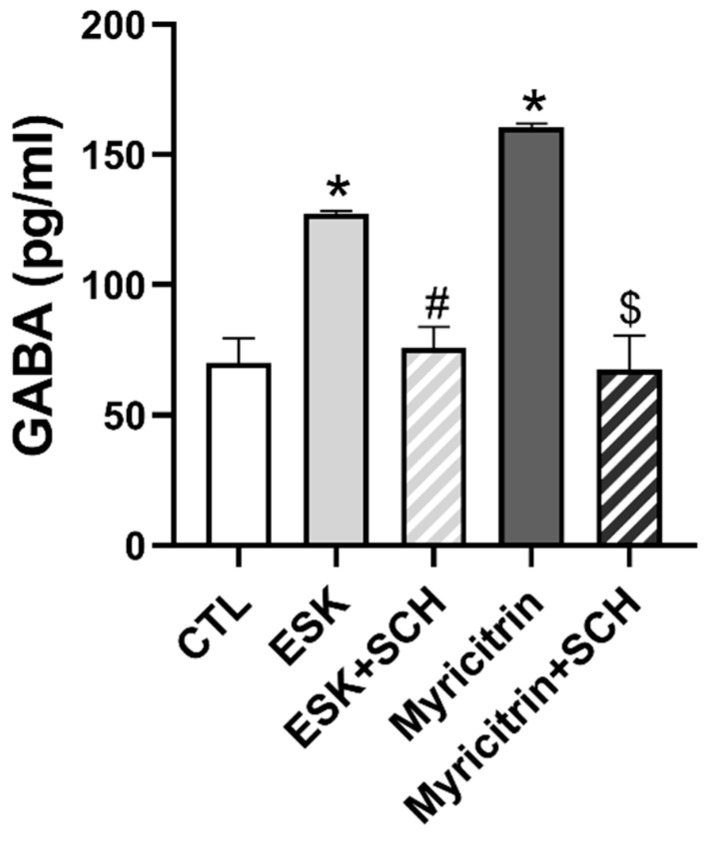
Effect of ESK and myricitrin on the level of GABA in the hypothalamus of mice. ESK (30 mg/kg, p.o.) and Myricitrin (10 mg/kg, p.o.) were administered 1 h before brain extraction. SCH (5 mg/kg, p.o.) was administered 15 min before ESK and myricitrin. Data are presented as Mean ± SEM (*n* = 3). * *p* < 0.05 vs. CTL; # *p* < 0.05 vs. ESK; $ *p* < 0.05 vs. Myricitrin. CTL, control.

To determine whether GABA is released upon stimulation of GABA neurons in the VLPO area after ESK and myricitrin administration, the GABA content was assessed using ELISA. The administration of ESK and myricitrin significantly increased the level of GABA; however, this increase was reversed by the A_2A_R antagonist, SCH (Figure 8). These results suggest that ESK and myricitrin activate GABA neurons in the VLPO and increase GABA release by activating A_2A_R.

## 4. Discussion

To our knowledge, this study is the first to elucidate the hypnotic effects of ESK and one of its active compounds, myricitrin, using pentobarbital-induced sleep experiments. Based on IHC, ESK and myricitrin augmented neuronal activity in the sleep-promoting regions. These effects were notably blocked by an A_2A_R antagonist. These findings indicate that the hypnotic effects of ESK and myricitrin may involve A_2A_R activation.

Adenosine influences neuronal activity regulating sleep and wakefulness, with levels increasing during wakefulness and decreasing during sleep [29,30,31]. The administration of adenosine has been shown to exert sedative and sleep-promoting effects [32,33]. Extracellular adenosine enhances adenylyl cyclase activity, stimulating sleep-active neurons via A_2A_R to induce sleep [34]. A_2A_R is expressed in GABAergic neurons within the VLPO, a critical area for sleep maintenance [35]. A_2A_R binds to Gs and activates adenylyl cyclase, increasing the cAMP concentration. Accordingly, PKA is activated, and CREB is phosphorylated [36]. Administration of the A_2A_R agonist, CGS21680, has been shown to promote sleep by increasing both non-rapid eye movement (NREM) and REM sleep, enhancing delta power during NREM sleep, and increasing c-Fos immunoexpression in GABAergic neurons in the VLPO [30,37]. According to our findings, ESK directly binds to A_2A_R and its hypnotic effect is significantly attenuated by SCH, an A_2A_R antagonist. Therefore, the hypnotic action of ESK might be mediated by A_2A_R activation.

The equilibrium between sleep-promoting neurons, including the VLPO, and wake-promoting neurons, such as the BF and LH, is essential for regulating the sleep-wake cycle [13]. To ascertain the specific brain areas involved in regulating sleep and contributing to the hypnotic effect of ESK, we assessed neuronal activity in the VLPO of mice using IHC. The administration of ESK increased c-Fos immunoexpression in the VLPO. These effects were inhibited by the A_2A_R antagonist, SCH. The VLPO, situated in the hypothalamus, is known to promote sleep, and the activation of GABAergic neurons directly induces sleep. Several studies have demonstrated that A_2A_R activation directly activates GABAergic neurons in the VLPO. Administration of the A_2A_R agonist, CGS21680, in the subarachnoid space adjacent to the VLPO, was found to inhibit the arousal system, induce sleep, and increase NREM sleep [38]. Furthermore, the intracerebroventricular administration of CGS21680 activated VLPO GABAergic neurons and promoted sleep. Previously, the intracerebroventricular administration of an A_2A_R antagonist was found to suppress c-Fos activity in VLPO GABAergic neurons [30]. The signaling pathway of A_2A_R involves Gs-coupled excitatory signaling [36]. Thus, the sleep-promoting function of adenosine via A_2A_R is believed to occur through excitatory signaling in sleep-promoting GABAergic neurons in the VLPO. GABA secretion in the VLPO is enhanced during slow-wave sleep and is reduced during arousal [39]. Using ELISA, we confirmed that the administration of ESK in the hypothalamus of mice increased GABA secretion.

Myricitrin, one of the active compounds of ESK, is a polyphenolic hydroxyflavonoid with various biological activities, including antibacterial, anti-inflammatory, anti-invasive, antioxidant, anxiolytic, antimanic, and antidepressant effects [40,41,42,43,44]. Recent studies have indicated that flavonoids, such as luteolin and quercetin, exhibit sedative and hypnotic effects [45,46]. Although myricitrin is structurally similar to luteolin and quercetin, data on its hypnotic effects have not been published [47]. In this study, we observed for the first time the hypnotic effects of myricitrin using a pentobarbital-induced sleep test. Myricitrin increased c-Fos neuronal activity in the VLPO and GABA release. In conclusion, we propose that the hypnotic effects of ESK and myricitrin are linked to the stimulation of sleep-promoting neurons. In addition, these hypnotic effects may be related to the activation of A_2A_R.

Based on our findings, ESK has novel activity as an A_2A_R agonist, exerting hypnotic effects in mice. Although A_2A_R primarily regulates GABAergic neurons in the VLPO, it can also modulate neural activity in wake-promoting regions such as the tuberomammillary nucleus and lateral hypothalamus. Therefore, further investigation is needed to determine whether ESK affects the activity of these wake-promoting neurons. Moreover, as the permeability of ESK extracts through the blood–brain barrier (BBB) is unknown, further investigations are needed to determine whether ESK extracts can cross the BBB.

## 5. Conclusions

Overall, our findings indicate that ESK and one of its active compounds, myricitrin, exhibit hypnotic effects. Activation of A_2A_R, which results in the stimulation of GABA neurons in the VLPO region, is proposed as the mechanism of sleep promotion. Altogether, ESK and myricitrin may serve as novel sleep aids for improving sleep.

## Data Availability

Data are contained within the article.
